# Sodium-glucose co-transporter-2 inhibitors in patients treated with immune checkpoint inhibitors

**DOI:** 10.1186/s40959-023-00199-6

**Published:** 2024-01-11

**Authors:** Moran Gvili Perelman, Rafael Y. Brzezinski, Barliz Waissengrin, Yasmin Leshem, Or Bainhoren, Tammi Arbel Rubinstein, Maxim Perelman, Zach Rozenbaum, Ofer Havakuk, Yan Topilsky, Shmuel Banai, Ido Wolf, Michal Laufer-Perl

**Affiliations:** 1 Division of Cardiology, Tel-Aviv Sourasky Medical Center, Tel Aviv, Israel; 2 Division of Oncology, Tel-Aviv Sourasky Medical Center, Tel Aviv, Israel; 3 School of Medicine, Tel Aviv University, Tel Aviv, Israel; 4 Internal Medicine T, Chaim Sheba Medical Center, Ramat-Gan, Israel; 5 Tulane University, New Orleans, LA USA

**Keywords:** ICIs, SGLT2, Cardio-oncology, Cardiotoxicity, Immune checkpoint inhibitor, Diabetes

## Abstract

**Background:**

Immune checkpoint inhibitors (ICIs) have revolutionized the prognosis of cancer. Diabetes mellitus (DM) has been shown to have a negative effect on patients treated with ICIs. Sodium-glucose cotransporter 2 inhibitors (SGLT2i) are effective antidiabetic therapies associated with reduced all-cause mortality and cardiovascular (CV) outcomes.

**Objective:**

To evaluate the prognostic value of SGLT2i on all-cause mortality and cardiotoxicity among patients treated with ICIs.

**Methods:**

We performed a retrospective analysis of patients diagnosed with cancer and type 2 DM (DM2) and treated with ICIs at our center. Patients were divided into two groups according to baseline treatment with or without SGLT2i. The primary endpoint was all-cause mortality and the secondary endpoint was MACE, including myocarditis, acute coronary syndrome, heart failure, and arrhythmia.

**Results:**

The cohort included 119 patients, with 24 (20%) patients assigned to the SGLT2i group. Both groups exhibited a comparable prevalence of cardiac risk factors, although the SGLT2i group displayed a higher incidence of ischemic heart disease. Over a median follow-up of 28 months, 61 (51%) patients died, with a significantly lower all-cause mortality rate in the SGLT2i group (21% vs. 59%, *p* = 0.002). While there were no significant differences in MACE, we observed zero cases of myocarditis and atrial fibrillation in the SGLT2i, compared to 2 and 6 cases in the non-SGLT2i group.

**Conclusions:**

SGLT2i therapy was associated with a lower all-cause mortality rate in patients diagnosed with cancer and DM2 and treated with ICIs. Further studies are needed to understand the mechanism and evaluate its benefit on cardiotoxicity.

**Graphical Abstract:**

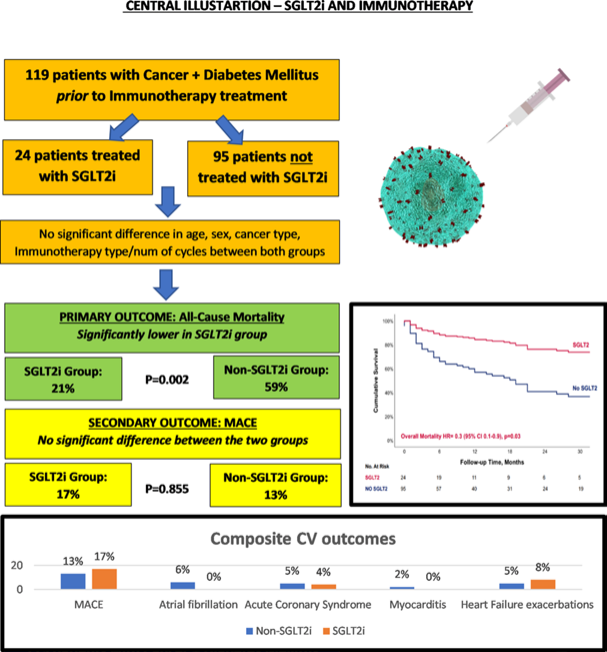

## Introduction

Tumor cells can avoid the host immune response and allow cell proliferation and metastasis [1]. Immune checkpoint inhibitors (ICIs) have revolutionized the prognosis of various types of cancer [2] by stimulating the host’s immune system to target tumor cells [3], thus leading to reductions in the tumor's ability to evade the host’s defense mechanisms and thereby hindering tumor cell survival. Accordingly, the use of ICIs therapy is increasing, both for metastatic disease and in earlier disease settings [4–6], with nearly 50% of patients with cancer eligible for ICIs therapy [7]. Several ICIs therapies are currently approved by the Food and Drug Administration (FDA), including programmed death-1 inhibitor (PD-1), programmed cell death-ligand 1 inhibitor (PD-L1), and cytotoxic T-lymphocyte–associated protein 4 (CTLA-4 [8]).

The response to ICIs therapy is diverse among the population, and while most patients will greatly benefit from the therapy, others will not. Therefore, there is a worldwide scientific focus to discover predisposing factors that may influence the response and prognosis of patients planned for ICIs therapy [9]. One of these commonly researched factors is Diabetes mellitus (DM)—which has been shown to have a negative effect on overall (OS) and progression-free survival (PFS) in patients treated with ICIs [10].

Moreover, the stimulation of the immune system may lead to inappropriate activation of pro-inflammatory T cells, which may infiltrate different organs, causing damage to the organs and leading to significant morbidity and mortality and the premature discontinuation of efficacious cancer therapy [11, 12]. ICIs-induced cardiotoxicity are relatively rare complication, however, potentially fatal [13]. While myocarditis is the most studied complication, other manifestations include acute coronary syndrome (ACS), heart failure (HF), and arrhythmias [12].

Sodium-glucose cotransporter 2 inhibitors (SGLT2i) are effective antidiabetic therapies in patients with type 2 DM (DM2) and are associated with improved glycemic control, reductions in body mass, improved blood pressure, and reduced overall mortality and CV outcomes [14, 15]. Recently, the beneficial effects of SGLT2i have been shown to improve cardiac outcomes and OS in patients with cancer treated with anthracyclines [16], as well as among a broad range of cancer therapies [17]. Currently, there is no data regarding the potential benefit of SGLT2i among patients diagnosed with DM2 and treated with ICIs therapy.

Our study aimed to evaluate the prognostic value of baseline therapy with SGLT2i among patients diagnosed with cancer and DM2, treated with ICIs therapies, on the development of all-cause mortality and ICIs-induced cardiotoxicity.

## Methods

### Study population and protocol

We conducted a retrospective, single-center, observational study at Tel-Aviv Sourasky Medical Center, a tertiary cancer center in Israel.

Consecutive medical records of all patients diagnosed with cancer and DM2, and treated with ICIs therapy were reviewed. Exclusion criteria included age of less than 18 years.

Patients were divided into two groups: patients treated with SGLT2i prior to ICIs therapy initiation – the SGLT2i group and patients not treated with SGLT2i – the non-SGLT2i group.

The study was approved by the local Helsinki regulatory ethics committee (Identifier: TLV-0228–16)

### Data collection

Data including baseline medical history and medications, malignancy status, ICIs therapy, previous chemotherapy therapy, blood tests, and echocardiography findings were obtained from health records. PFS was determined through a review of the electronic medical charts by an oncologist (B.W and O.B.).

### Study endpoints

The primary endpoint was all-cause mortality, extracted from patient charts and the population registry bureau.

The secondary endpoint was major adverse cardiovascular events (MACE), defined as the composite of myocarditis, ACS, HF exacerbation (including HF hospitalizations or emergency room visits due to either denovo or acute on chronic HF diagnosis), and arrhythmia (including atrial fibrillation (AF), atrial flutter, ventricular tachycardia and ventricular fibrillation). These endpoints were chosen, as they are considered as ICIs-induced cardiotoxicity [8]. The diagnosis of each endpoint was determined through a review of the electronic medical charts by a cardio-oncologist (M.L.P), according to the accepted European Society of Cardiology (ESC) guidelines.

### Statistical analysis

All continuous variables are displayed as mean (± standard deviation (SD)) for normally distributed variables, or median [interquartile range (IQR)] for variables with nonnormal distributions. Categorical variables are displayed as the number (%) of individuals within each group.

Continuous variables were compared by a two-tailed unpaired t-test for normally distributed variables and by the Mann‐Whitney U test for non‐normally distributed ones. To assess associations among categorical variables, we used a Chi-square test. The median follow-up time for all-cause mortality was calculated using the reverse Kaplan-Meir method [18].

All-cause mortality was evaluated using univariate and multivariable Cox proportional hazard regression. Cumulative survival curves divided by SGLT2i treatment status are presented. We adjusted our model for age, gender, cancer type, cancer stage, protocol therapy, conventional risk factors (hypertension, dyslipidemia, prior ischemic heart disease (IHD), and obesity), and use of medications (statins and renin–angiotensin–aldosterone system inhibitors (RAASi)).

PFS between the groups was analyzed using Kaplan–Meier methods and the log-rank test.

A two-tailed *p* < 0.05 was considered statistically significant. All analyses were performed with the SPSS (IBM SPSS Statistics, version 28, IBM Corp., Armonk, NY, USA, 2016), The R statistical package (version 3.3.1) (R Foundation for Statistical Computing, Vienna, Austria), and GraphPad Prism version 9.00 (GraphPad Software, La Jolla, CA, USA).

## Results

### Baseline patient characteristics

From November 2015 to August 2022, 119 patients with cancer and the diagnosis of DM2, prior to the initiation of ICIs therapy, were identified and included in our cohort. Overall, the SGLT2i group included 24 (20%) patients and the non-SGLT2i group included 95 patients.

Baseline clinical characteristics are summarized in Table 1. Our cohort’s mean age was 71 ± 10 years and was predominantly male (62%). There were no significant differences in age, gender, and body mass index (BMI) between the two groups.

**Table 1:** Baseline Characteristics of the cohort population

	**Entire Cohort**	**non-SGLT2i**	**SGLT2i**	***p* value**
**Number**	119	95	24	
**Demographics**				
Age, years	71 (10)	71 (11)	70 (6)	0.578
Female, N (%)	45 (38)	40 (42)	5 (21)	0.092
**Cancer Types,** N (%)				0.065
NSCLC	29 (24)	24 (25)	5 (21)	
Melanoma	19 (16)	17 (18)	2 (8)	
Renal Cell Carcinoma	27 (23)	21 (22)	6 (25)	
Hepatocellular Carcinoma	23 (19)	17 (18)	6 (25)	
Breast	6 (5)	2 (2)	4 (17)	
Cervical Squamous	6 (5)	6 (6)	0 (0)	
Other	9 (8)	8 (8)	1 (4)	
**Cancer Stages** N (%)				0.851
2	1 (1)	1 (1)	0 (0)	
3	13 (11)	10 (11)	3 (12)	
4	105 (88)	84 (88)	21 (88)	
**Metastasis** N (%)	105 (93)	83 (93)	22 (92)	1.000
**Brain metastasis** N (%)				0.507
No	31 (27)	23 (26)	8 (33)	
Yes	11 (10)	10 (11)	1 (4)	
Unknown	71 (63)	56 (63)	15 (63)	
**Bone metastasis** N (%)				0.159
No	64 (57)	52 (59)	12 (50)	
Yes	41 (37)	29 (33)	12 (50)	
Unknown	7 (6)	7 (8)	0 (0)	
**Lung metastasis** N (%)				0.360
No	49 (44)	38 (43)	11 (48)	
Yes	48 (43)	37 (42)	11 (4)	
Unknown	15 (13)	14 (16)	1 (4)	
**Liver metastasis** N (%)				0.358
**No**	60 (53)	46 (52)	14 (58)	
**Yes**	46 (41)	36 (40)	10 (42)	
Unknown	7 (6)	7 (8)	0 (0)	
**Lymph node metastasis** N (%)				0.189
No	33 (30)	25 (28)	8 (33)	
Yes	68 (61)	52 (59)	16 (67)	
Unknown	11 (10)	11 (13)	0 (0)	
**ECOG** N (%)				0.094
0	36 (41)	24 (34)	12 (71)	
1	39 (44)	35 (49)	4 (4)	
2	9 (10)	8 (11)	1 (6)	
3	3 (3)	3 (4)	0 (0)	
**Immunotherapy Type** N (%)				0.441
Pembrolizumab	28 (24)	24 (25)	4 (17)	
Nivolumab	10 (8)	8 (8)	2 (8)	
Avelumab	9 (8)	8 (8)	1 (4)	
Atezolizumab	36 (30)	25 (26)	11 (46)	
Ipilimumab + Nivolumab	36 (30)	30 (32)	6 (25)	
**Previous ICIs** N (%)	4 (3)	3 (3)	1 (4)	0.713
**Number of ICIs cycles**, median [IQR]	6 [3, 17]	5 [3, 17]	8 [4, 14]	0.261
**Number of ICIs lines,** median [IQR]	1 [1, 2]	1 [1, 2]	1 [1, 1]]	0.201
**Line of treatment** N (%)				0.319
1	72 (65)	55 (63)	17 (71)	
2	31 (28)	26 (30)	5 (21)	
3	6 (5)	5 (6)	1 (4)	
4	1 (1)	1 (1)	0 (0)	
5	1 (1)	0 (0)	1 (4)	
**Previous lines,** median [IQR]	0 [0,1]	0 [0, 1]	0 [0,0]	0.595
0	87 (73)	67 (71)	20 (83)	
1	21 (18)	19 (20)	2 (8)	
2	6 (5)	5 (5)	1 (4)	
3	3 (3)	2 (2)	1 (4)	
4	2 (2)	2 (2)	0 (0)	
**Chemotherapy/Biological plus ICIs** N (%)	39 (33)	23 (24)	16 (67)	** < 0.001**
Only ICIs, N (%)	80 (67)	72 (76)	8 (33)	
Chemo plus ICIs**,** N (%)	24 (20)	13 (14)	11 (46)	
Biological plus ICIs**,** N (%)	15 (13)	10 (11)	5 (21)	
**Cardio-Vascular Risk Factors**				
Hypertension, N (%)	70 (59)	56 (59)	14 (58)	1.000
Smoking, N (%)	9 (8)	7 (7)	2 (8)	1.000
Ischemic Heart Disease, N (%)	26 (22)	16 (17)	10 (42)	**0.019**
Obstructive Sleep Apnea, N (%)	1 (1)	1 (1)	0 (0)	1.000
BMI baseline, mean (SD)	28.6 (5.5)	28.7 (5.7)	28.3 (4.8)	0.708
Hyperlipidemia, N (%)	41 (34)	29 (31)	12 (50)	0.120
Chronic Kidney Disease, N (%)	8 (7)	6 (6)	2 (8)	1.000
**Cardiovascular Medications,** N (%)				
MRA	11 (9)	6 (6)	5 (21)	0.072
ARNI	4 (3)	2 (2)	2 (8)	0.380
ACEI	41 (34)	30 (32)	11 (46)	0.283
ARB	35 (29)	26 (27)	9 (38)	0.470
Statin	82 (69)	60 (63)	22 (92)	**0.014**
Furosemide	17 (14)	12 (13)	5 (21)	0.484
Beta blocker	58 (49)	43 (47)	15 (62)	0.200
Metformin	91 (76)	71 (75)	20 (83)	0.375
GLP-1	1 (1)	1 (1)	0 (0)	1.000
**Baseline Laboratory Parameters**				
HbA1C %, median [IQR]	6.6 [6.1, 7.3]	6.6 [6.0, 6.9]	7.1 [6.3, 8.3]	0.089
WBC K/Ul, mean (SD)	9.4 (3.4)	9.5 (3.5)	9.2 (3.0)	0.756
Platelet K/Ul, mean (SD)	240.2 (83.7)	243.8 (85.1)	225.6 (77.6)	0.352
Hematocrit %**,** mean (SD)	33.6 (6.2)	32.8 (6.0)	37.0 (6.1)	**0.003**
Creatinine mg/Dl, median (IQR)	0.9 [0.7, 1.2]	0.9 [0.7, 1.2]	1.0 [0.9, 1.2]	0.351
Troponin I ng/L baseline, median (IQR)	5.5 [2.5, 14.2]	6.0 [4.0, 15.0]	3.0 [0.5, 7.5]	0.056
Troponin I ng/L following ICIs, median (IQR)	8.0 [5.0, 23.0]	8.0 [5.0, 23.5]	8.0 [5.0, 21.0]	0.762

*SGLT2I* Sodium-glucose cotransporter 2 inhibitors, *N* number, *NSCLC* Non-small cell lung cancer, *SCC* squamous cell carcinoma, *ECOG* Eastern Cooperative Oncology Group, *ECOG 0* Fully active, *ECOG1* Restricted in physically strenuous activity, *ECOG2* Ambulatory and capable of all selfcare but unable to carry out any work activities, *ECOG3* Capable of only limited selfcare, confined to bed or chair more than 50% of waking hours, *ICIS* Immune checkpoint Inhibitors, *SD* Standard deviation, *IQR* interquartile range, *BMI* body mass index, *MRA* Aldosterone receptor antagonists, *ARNI* angiotensin receptor neprilysin inhibitor, *ACEI* Angiotensin-converting enzyme, *ARB* Angiotensin receptor blockers, *GLP-1A* Glucagon-like peptide-1 agonist, *WBC* white blood cell

Cancer types were similar between the SGLT2i and non-SGLT2i groups. The most common cancer diagnosis was non-small cell lung cancer (NSCLC), renal cell carcinoma (RCC), and hepatic cell carcinoma (HCC) in both groups, with most patients classified as stage 4 (88%). While data regarding the burden of disease (brain, liver, bone, and lymph node metastasis) or Eastern Cooperative Oncology Group (ECOG) stage was not available in all patients, no significant differences were observed between the groups (Table 1).

The specific cancer therapy protocol was chosen by the treating oncologist according to current best practices. ICIs therapies included pembrolizumab (anti-PD-1) (24%), nivolumab (anti-PD-1) (8%), avelumab (anti-PD-L1) (8%), atezolizumab (anti-PD-L1) (30%) and combined ipilimumab (anti-CTLA-4) + nivolumab (30%) [8]. The median number of cycles was 6 [3, 17]. No significant differences were observed between the groups regarding the type of ICIs therapy, single or combined ICIs, the number of cycles, and the number of ICIs lines (Table 1). Overall, 39 (33%) patients were treated with a combined protocol therapy of ICIs and chemotherapy / biological therapy, with a significantly higher prevalence in the SGLT2i group (67% vs. 24%, *p* < 0.001) (Table 1).

There were 2 types of SGLTi therapies with the most common being empagliflozin (83%), followed by dapagliflozin (17%). No significant differences were observed regarding the use of metformin (*p* = 0.375) between the groups and only one patient was treated with a glucagon-like peptide-1 (GLP-1) agonist. Baseline hemoglobin A1c (HbA1c) was available only in 43 patients with a higher trend in the SGLT2i group (7.1 [IQR 6.3, 8.3] vs. 6.6 [IQR 6.0, 6.9], *p* = 0.089).

Both groups had similar prevalence of cardiac risk factors (HTN, hyperlipidemia, smoking, and chronic kidney disease (CKD)). However, a history of IHD was significantly higher in the SGLT2i group (42% vs. 17%, *p* = 0.019). While statin therapy was significantly higher among the SGLT2i group (92% vs. 63%, *p* = 0.014), no significant differences were observed regarding cardio-protective therapy, including RAASi, aldosterone receptor antagonist (MRA) and beta blockers (BB) (Table 1).

Overall, 46 patients (38%) had a baseline echocardiography assessment, with a trend for lower mean left ventricle ejection fraction (LVEF) in the SGLT2i group (50 ± 16% vs. 57 ± 9%, *p* = 0.064) and 60 patients (50%) performed baseline high-sensitivity troponin I level, which was within the normal range (≤ 50 nanogram/Liter (ng/L)), with a trend for lower levels in the SGLT2i group (3.0 [0.5, 7.5]ng/L vs. 6.0 [4.0, 15.0]ng/L, *p* = 0.056).

Baseline hematocrit (HCT) was significantly higher in the SGLT2i group (37 ± 6% vs. 33 ± 6%, *p* = 0.003). Other baseline labs including platelets, white blood cells, and creatinine were similar between both groups.

### Primary endpoint

**Fig. 1:**
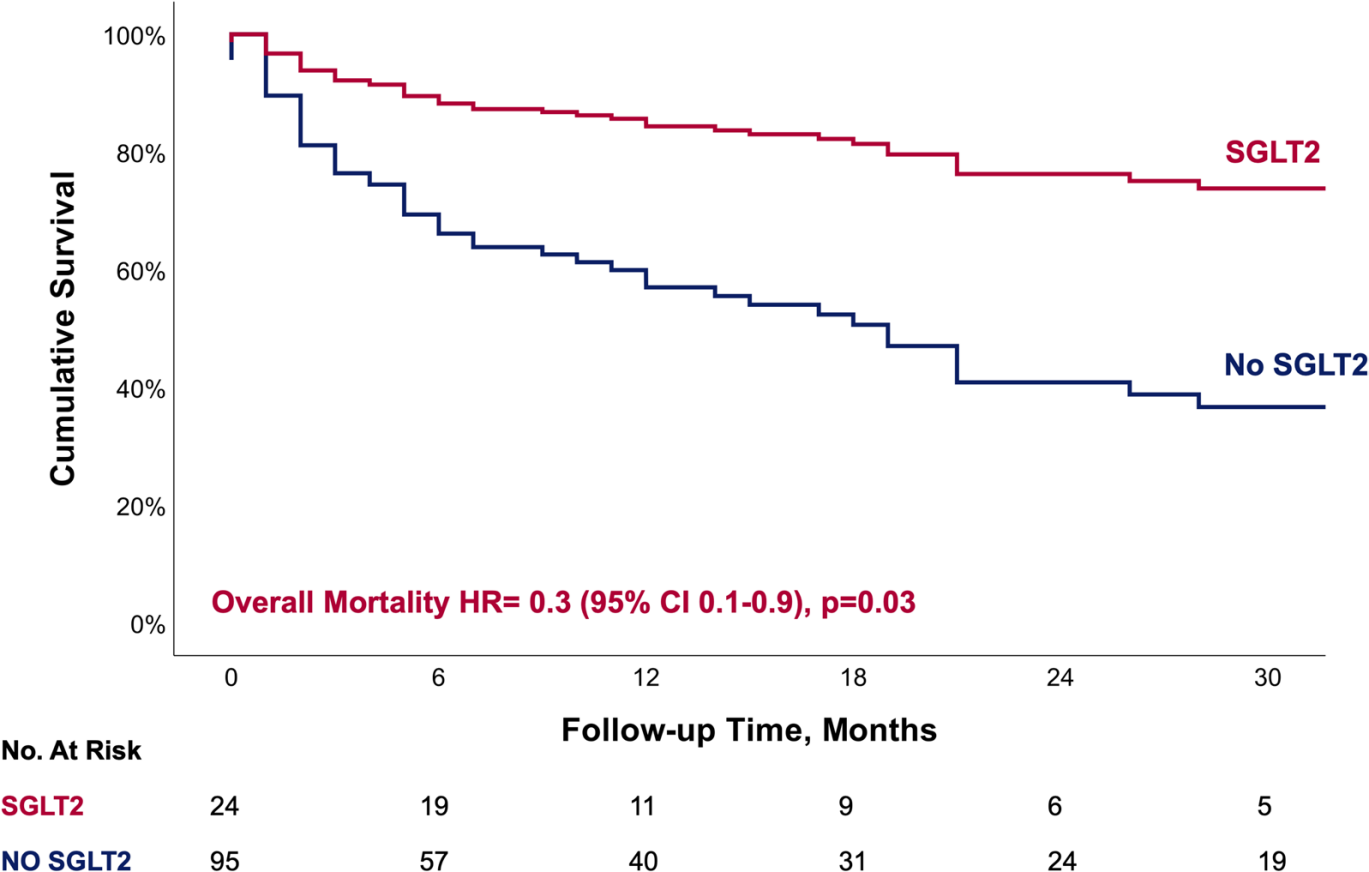
Cox regression curve for overall mortality

Over a median follow-up of 28 [IQR 10, 43] months, 61 (51%) patients died, with a significantly lower all-cause mortality among the SGLT2i group (21% vs. 59%, *p* = 0.002) (Table 2). Using a multivariable cox regression analysis that included all baseline characteristics showing significant differences between the groups, or having a possible clinical significance (Table 3), SGLT2i emerged as an independent significant predictor for lower all-cause mortality (Hazard Ratio (HR) 0.300 (95% CI 0.104–0.865), *p* = 0.026) (Fig. 1). While combined protocol with chemotherapy did not emerge as a significant predictor for all-cause mortality, combined biological therapy had a protective value with lower mortality.

**Table 2:** Primary and secondary endpoints

	Entire Cohort	Non-SGLT2i	SGLT2i	*p* value
**All-cause Mortality, N (%)**	**61 (51)**	**56 (59)**	**5 (21)**	**0.002**
Progression, N (%)	96 (81)	78 (82)	18 (75)	0.431
MACE, N (%)	16 (13)	12 (13)	4 (17)	0.855
Arrhythmia Composite, N (%)	6 (5)	6 (6)	0 (0)	0.458
Av block, N (%)	1 (1)	1 (1)	0 (0)	1.000
Atrial fibrillation, N (%)	6 (5)	6 (6)	0 (0)	0.458
Acute Coronary Syndrome, N (%)	6 (5)	5 (5)	1 (4)	1.000
Myocarditis, N (%)	2 (2)	2 (2)	0 (0)	1.000
Heart Failure admissions, N (%)	2 (2)	1 (1)	1 (4)	0.864
Heart Failure exacerbations, N (%)	7 (6)	5 (5)	2 (8)	0.932

*SGLT2i* Sodium-glucose cotransporter 2 inhibitors, *N* number, *MACE* Major adverse cardiovascular events

**Table 3:** Cox proportional hazards regression for all-cause mortality

	**Hazard Ratio**	**95.0% CI**		***P* value**
		Lower	Upper	
**SGLT-2 (yes/no)**	0.300	0.104	0.865	**0.026**
Age	1.016	0.983	1.050	0.338
Gender	0.494	0.241	1.014	0.054
**Cancer Types**				**0.030**
NSCLC	0.583	0.214	1.588	0.291
Melanoma	0.401	0.165	0.979	**0.045**
Renal Cell Carcinoma	2.716	1.024	7.202	**0.045**
Hepatocellular Carcinoma	3.515	0.617	20.044	0.157
Breast	1.092	0.264	4.520	0.903
Cervical Squamous	1.180	0.355	3.925	0.787
**Chemo**therapy/Biological**_plus_I**CIs				0.115
Chemo_plus_ICIs	0.770	0.301	1.967	0.584
Biological_plus_ICIs	0.247	0.066	0.923	**0.038**
**Cancer Stage**				0.175
Stage at immuno Start(1)	0.450	0.031	6.517	0.559
Stage at immuno Start(2)	1.565	0.152	16.118	0.706
Hypertension	1.112	0.543	2.275	0.772
Ischemic Heart Disease-Baseline	0.713	0.308	1.648	0.429
Obesity	1.717	0.407	7.238	0.461
Hyperlipidemia	0.537	0.257	1.122	0.098
Chronic Kidney Disease	.768	.244	2.423	0.653
Statin	1.720	.814	3.634	0.155
RAASi treatment	.898	.445	1.813	0.765

^*^Stage 2 for reference

Only 1 patient was classified as CV mortality and included in the non-SGLT2i group.

### Secondary endpoints

**Fig. 2:**
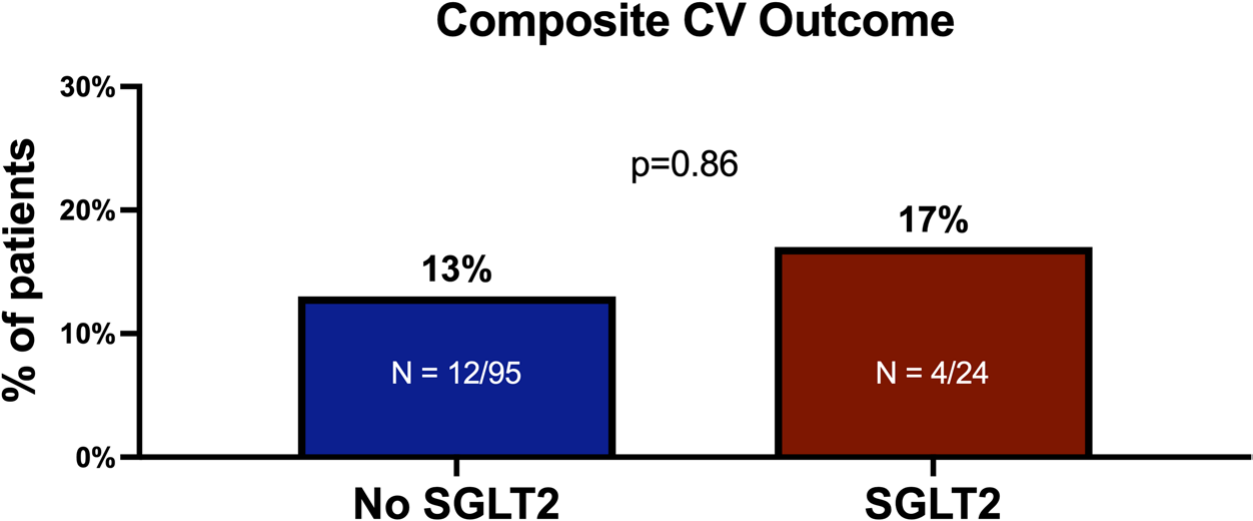
Composite MACE

Overall, 16 (13%) patients developed MACE during follow-up, 4 in the SGLT2i groups vs. 12 in the non-SGLT2i group. There were no significant differences in the composite of MACE between the SGLT2i and the non-SGLT2i groups (17% vs. 13%, *p* = 0.855) (Fig. 2). Evaluating each parameter, we observed a higher non-significant incidence of atrial fibrillation (AF) (6% vs. 0%, *p* = 0.458) and myocarditis (2% vs. 0%, *p* = 1.000) (Table 2) among the non-SGLT2i group, while higher incidence of HF exacerbation was observed among the SGLT2i group (8% vs. 5%, *p* = 0.932).

**Fig. 3:**
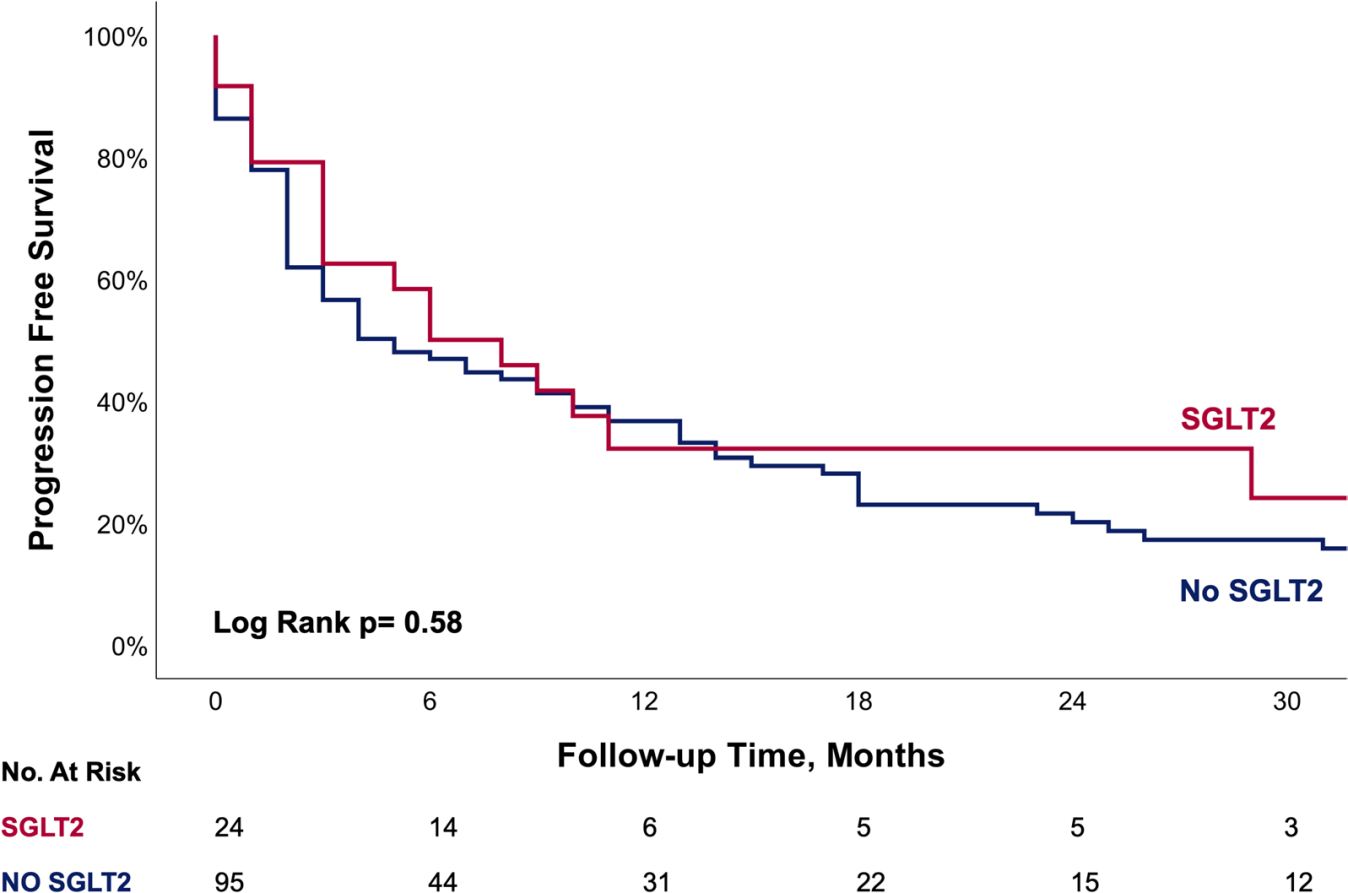
Kaplan–Meier curve showing Progression free survival between patients with and without SGLT2 treatment

No significant differences were observed in PFS between the two groups (Fig. 3).

## Discussion

In our cohort, including patients diagnosed with cancer and DM2, and treated with ICIs therapy, baseline treatment with SGLT2i emerged as an independent strong prognosis predictor, with a 70% reduction in all-cause mortality compared to patients without SGLT2i. While no significant differences were observed regarding the incidence of MACE between the groups, patients treated with SGLT2i showed zero events of AF and myocarditis.

SGLT2i are effective antidiabetic therapies in patients diagnosed with DM2 and are associated with reduced mortality and CV outcome [14, 15], as shown in the EMPA-REG and DECLARE-TIMI 58 trials. The beneficial effects of SGLT2i have been proven to be well beyond glycemic control. The DAPA-HF trial showed that among patients with HF and a reduced LVEF, treatment with dapagliflozin reduced significantly the risk of worsening HF, CV death, and all-cause mortality compared to placebo, regardless of the presence or absence of DM2 [19]. In the EMEPROR-preserved trial, empagliflozin reduced the combined risk of CV death and hospitalization for HF in patients with HF and preserved LVEF, regardless of the presence or absence of DM2 [20].

Recent trials have shown that SGLT2i may also have a beneficial effect on patients diagnosed with cancer. Gongora et al [16] showed that SGLT2i was associated with a lower rate of all-cause mortality and cardiac events among patients with cancer and DM who were treated with anthracyclines, compared to a control group not treated with SGLT2i. Chiang et al [17] observed the impact of SGLT2i on HF and mortality in patients with various types of cancer receiving different treatments and found that the use of SGLT2i was associated with a lower rate of incident HF and with prolonged survival in patients with cancer and DM2. Similar to those trials, in our study, we found that baseline treatment with SGLT2i among patients treated with ICIs therapy played an independent strong predictor for lower all-cause mortality.

In general, DM2 was found to have a negative effect on survival and PFS in various types of cancer [21, 22]. One of the proposed theories is that hyperglycemia accelerates the progression of the tumor by enhancing the proliferation, migration, and invasion of tumor cells [23]. Jacobi et al [10] showed that patients with DM2 and metastatic NSCLC who were treated with ICIs therapy had a worse outcome in PFS and survival compared to patients without DM2. Furthermore—recently, DM2 has been shown to have a negative effect specifically on patients with NSCLC treated with Pembrolizumab [24].

Several studies have shown that SGLT2i can slow tumor growth in mouse models of breast, colon, gastrointestinal, lung, and liver tumors by promoting a fasting-like state and mitigating hyper-insulinoma [25], so it may be interesting to consider that the beneficial effect of SGLT2i on patients with cancer might be an anti-neoplastic one. Preclinical studies showed that SGLT-specific positron emission tomography tracers accumulate in tumor cells and patient-derived xenografts and were reduced by SGLT2i [26]. In our trial, the SGLT2i group had higher levels of HbA1C compared to the non-SGLT2i group, which reflects higher blood glucose levels/reduced glycemic control compared to the control group. Nonetheless, patients in the SGLT2i group had lower mortality rates, which strengthens the specific beneficial effect of SGLT2i treatment, beyond glycemic control, among patients with cancer.

Assessing the incidence of MACE, we did not find significant differences between the groups. This might be explained by the higher prevalence of baseline IHD among the SGLT2i group, and therefore the higher risk of developing MACE, compared to the non-SGLT2i group. Furthermore, we believe that ICI-induced cardiotoxicity is still underdiagnosed [27] by the treating physician, as the majority of patients treated with ICIs do not perform routine CV assessment with electrocardiogram, cardiac biomarkers, and echocardiography. With the new ESC 2022 cardio-oncology guidelines [8], this might change soon. Large prospective trials are needed. Due to the low number of CV events, we could not discuss the statistical differences in each parameter of MACE. However, we observed zero events of AF, compared to 6 events in the non-SGLT2i group. A lower incidence of AF has also been described in the SGTL2i trials among the general popultaion [28], and might have contributed to the lower mortality in the SGLT2i group. There are multiple possible mechanisms through which SGLT2i may reduce AF, including a reduction in body weight, blood pressure, and volume. Studies on animal models indicate that SGLT2i may reduce atrial fibrosis and adverse remodeling, in addition to improving cellular metabolisms and bioenergetics such as ion handling and mitochondrial function [29, 30]. A recently published study by Avula et al [31]. showed that diabetic oncologic patients with a diagnosis of cardiomyopathy or HF, due to cardio-toxic therapies showed improved CV outcomes, including HF exacerbations, when treated with SGLT2i. Contrary to those findings we did not observe a reduction in HF exacerbations in our study. These contradicting results can be explained by the difference in the cohort population. While the study by Avula et al [31] included only patients with a baseline diagnosis of cardiac dysfunction of HF, this was not an inclusion criterion in our trial. Given the fact that SGLT2i are the first line recommended therapy for patients with CV disease [14], we noticed a significantly higher prevalence of IHD among the SGLT2I group. Therefore, we should not be surprised by the higher incidence of HF exacerbations among patients with baseline IHD, compared to patients without. Even though, those differences did not reach a statistically significant.

While the protective mechanism of SGL2i is still unclear, one important parameter is considered to be an anti-inflammatory effect [32]. SGLT2i exerts anti-atherosclerotic properties attenuating inflammatory factors and reducing myocardial infarction, HF, and MACE in patients with DM2 [33]. As ICIs-induced cardiotoxicity is considered to be driven by the pro-inflammatory cytokine storm induced in myocardial tissues [34], the anti-inflammatory effect of SGLT2i may play a significant role in the prevention of ICIs-induced cardiotoxicity and mortality. On the other hand, the anti-inflammatory effect might diminish the efficacy of ICIs therapy. Trying to asses this concern, we observed no differences in PFS, implying that SGLT2i did not reduce the efficacy of ICIs therapy. Furthermore, patients in the SGLT2i group presented with lower all-cause mortality, which probably insinuates that this mechanism is more complex. In theory, it is possible that this anti-inflammatory effect could explain the absence of myocarditis diagnosis in this group, a fatal disease caused by overactivation of the immune system [13, 35], compared to the two diagnosed cases in the non-SGLT2i group. While statin therapy was more prevalent in the SGLT2i group and is known for its anti-inflammatory effect as well [36], it did not emerge as a significant independent predictor for all-cause mortality in a multivariable cox regression analysis.

Our finding is particularly intriguing in light of the recent paper by Cortellini [37], which revealed that the use of glucose-lowering medications, particularly metformin, among patients treated with ICIs, were found to be associated with increased mortality. Those finding may point out the specific beneficial mechanism of SGLT2i, beyond glucose-lowering.

Our study has several limitations. First, it is a single-center study and thus generalization of our results is limited. Second, this is a retrospective study; therefore, our results are subject to potential confounders that may be biased by its design. Additionally, because the data was collected retrospectively, there are notable gaps in information on metastatic spread and ECOG stage, therefore not allowing us to adjust for those parameters, aspects that could have had an impact on the outcomes. Third, the lack of routine assessment of cardiac biomarkers and echocardiography among the whole cohort population prevented us from assessing the real cardiotoxicity incidence in our trial. Last, we recognize that the relatively small sample size of the cohort, especially the treatment group, reduces our statistical results and therefore should be taken with caution. Larger prospective trials or meta-analysis are needed to establish our findings and to assess ICIs-induced cardiotoxicity.

## Conclusions

SGLT2i therapy was associated with a lower all-cause mortality rate in patients with cancer and DM2 treated with ICIs therapy, in addition to lower events of AF and Myocarditis. Further studies are needed to understand the mechanism and evaluate its benefit on CV outcomes.

## Data Availability

Data cannot be shared for ethical/privacy reasons.

## Abbreviations

ICI: Immune checkpoint inhibitors
SGLT2i: Sodium-glucose cotransporter 2 inhibitors
PFS: Progression-free survival
DM: Diabetes mellitus
IHD: Ischemic heart disease
